# Safety and effectiveness assessment of endovascular recanalization for non‐acute middle cerebral artery occlusion

**DOI:** 10.1111/cns.14426

**Published:** 2023-08-29

**Authors:** Zhang Xi, Duan Guangxin, Zhang He, Chen Zhibin, Luo Yun, Zhang Tingzheng, Xu Yun, Li Jingwei

**Affiliations:** ^1^ Department of Neurology, Medical School and The State Key Laboratory of Pharmaceutical Biotechnology, Drum Tower Hospital Nanjing University Nanjing China; ^2^ Jiangsu Province Stroke Center for Diagnosis and Therapy Nanjing China

**Keywords:** endovascular treatment, ischemic stroke, non‐acute MCA occlusion, recurrence of stroke

## Abstract

**Background:**

Endovascular treatment for patients with symptomatic nonacute middle cerebral artery occlusion remains clinically challenging, and proof of a beneficial effect on functional outcome is lacking. We aim to evaluate the effectiveness and safety of endovascular recanalization for patients with symptomatic nonacute middle cerebral artery occlusion.

**Methods:**

Ninety‐eight patients with symptomatic atherosclerotic nonacute middle cerebral artery occlusion were divided into drug treatment groups (42) and endovascular treatment groups (56). The rate of recanalization, peri‐procedural complications, and follow‐up results were evaluated.

**Results:**

Among the 56 patients who received endovascular treatment, 53 (94.6%) achieved successful recanalization. The rate of peri‐procedural complications was 7.1% (4/56), and the death rate was 1.8% (1/56). Any stroke within 90 days was 7.1% (4/56). Among the 42 patients in drug treatment group, any stroke within 90 days was 19.0% (8/42), death rate was 0.

**Conclusion:**

Among patients with symptomatic nonacute middle cerebral artery occlusion with a short length of occlusion and a moderate‐to‐good collateral circulation, endovascular treatment seems to be safe. And endovascular treatment could reduce the recurrence rate of stroke.

## Graphical abstract

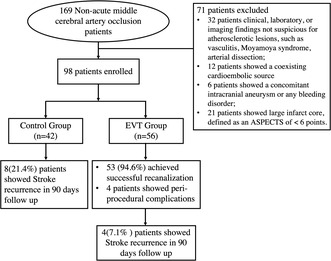

## 1 INTRODUCTION

Nonacute intracranial atherosclerotic occlusion (NAICAO) refers to the occlusion of large intracranial arteries for more than 24 h, which plays a key role in the occurrence of cerebral ischemic stroke.^1^, ^2^, ^3^ Among Asian stroke or TIA patients, the incidence of intracranial large artery occlusion was as high as 34.5%, while the incidence of intracranial large artery stenosis was 6.4%, and the incidence of extracranial artery disease (stenosis or occlusion) was 14.6%.^4^, ^5^ Patients with intracranial artery occlusion not only have more severe neurological dysfunction but also a higher recurrence rate of stroke or TIA.^6^, ^7^

The recurrence rate of stroke in patients with NAICAO is 3.6%–22.0%.^8^ Epidemiological investigation shows that 33%–50% of ischemic stroke and 50% of TIA in China are related to intracranial artery stenosis or occlusion, which is an extremely large patient group.^9^, ^10^

Several researches showed that the middle cerebral artery seemed more likely to occlude than other intracranial arteries.^11^ For patients with symptomatic nonacute middle cerebral artery occlusion (NAMCAO), the incidence of stroke was as high as 35.6%.^12^, ^13^, ^14^ For NAMCAO patients, drug therapy is in the dominant position. But 23.4% of patients still have strokes even with the best drug treatment.^15^ The surgical treatment for NAMCAO is intracranial and extracranial artery bypass grafting. However, several studies have proved that, compared with standard medical treatment, surgical bypass surgery did not reduce the recurrence of stroke caused by MCA occlusion.^16^, ^17^, ^18^ The effect of medical treatment is dissatisfying, and the efficacy of extracranial intracranial artery bypass grafting has not been finally proven in large studies. Endovascular therapy (balloon angioplasty or stenting) is also an inevitable choice for MCA occlusion. But endovascular treatment (EVT) is still in its infancy. Many stroke centers have tried endovascular treatment for these patients. Kangning Chen reported the results of EVT in 16 cases of nonacute intracranial large artery occlusion, and only 2 patients failed to recanalize.^19^ Ning Ma reported 2 cases of symptomatic NAMCAO patients treated with balloon angioplasty and stent implantation, and the success rate of the operation was 100%.^20^

Endovascular treatment for patients with symptomatic nonacute middle cerebral artery occlusion remains clinically challenging, and proof of a beneficial effect on functional outcome is lacking. We aim to evaluate the effectiveness and safety of endovascular recanalization for patients with symptomatic nonacute middle cerebral artery occlusion.

## 2 METHODS

### 2.1 Patients

**FIGURE 1:**
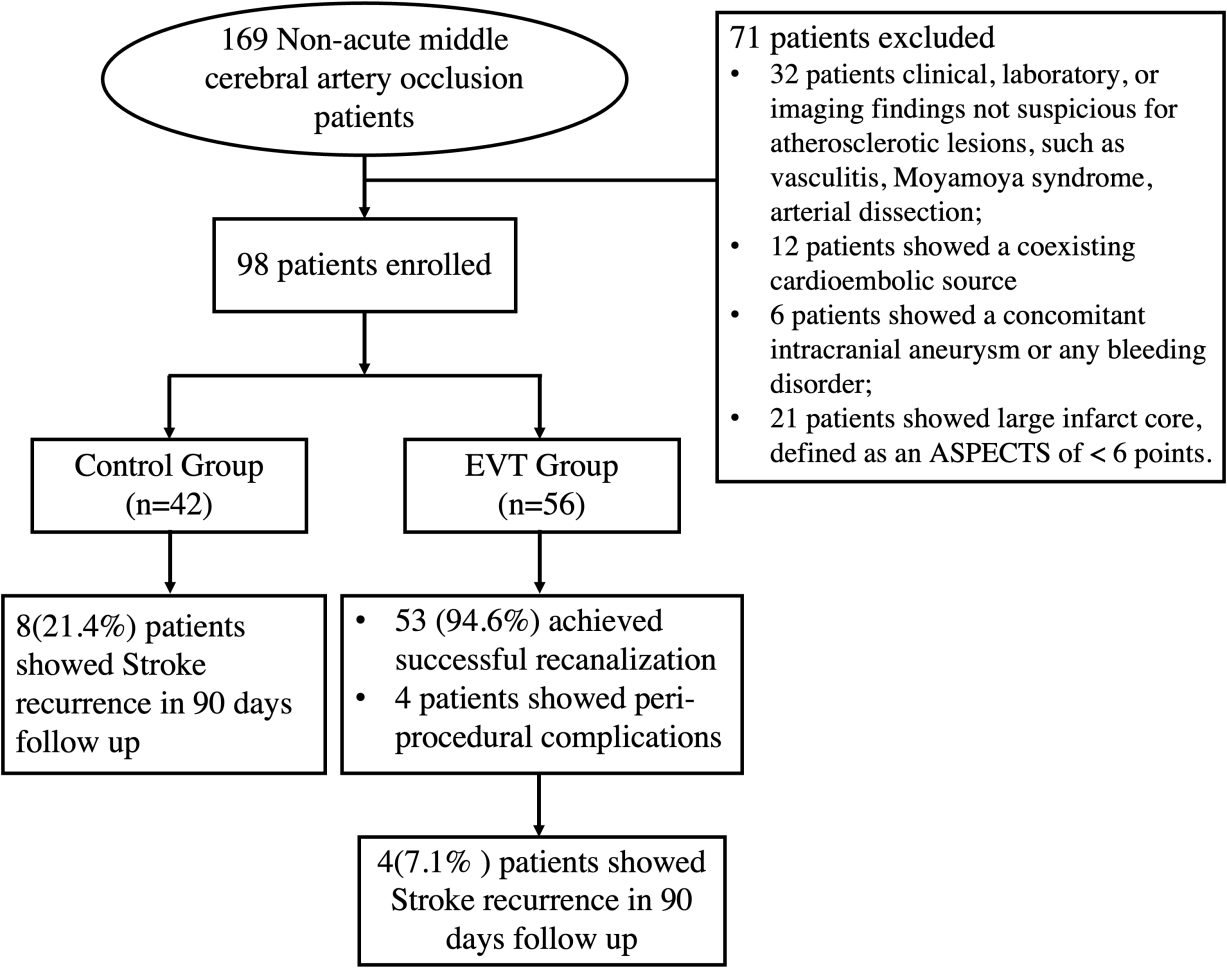
Flow chat of the study.

This is a retrospective study. A total of 169 stroke patients presented with nonacute middle cerebral artery occlusion February 2018 to April 2021, and 98 were enrolled. A flowchart of the study is offered in Figure 1. The institutional review board approved this study, and the IRB (Ethics Committee of Nanjing Drum Tower Hospital) number: 2021‐399‐02.

Selection criteria for NAMCAO patients include: (1) MCA occlusion was confirmed by digital subtraction angiography (DSA), and occlusion was defined as no forward flow; (2) presenting with an occluded artery‐related stroke; (3) time of onset exceeds 1 month and is less than 3 months; (4) occlusion length less than 2 cm (measured by high‐resolution vessel wall); (5) decreased perfusion in the occluded arterial supply area (measured by magnetic resonance perfusion); (6) ASITN/SIR ≥3 (measured by DSA); (7) a preoperative mRS score ≤3; (8) patient refusal to undergo bypass surgery. The exclusion criteria were as follows: (1) clinical, laboratory, or imaging findings not suspicious for atherosclerotic lesions, such as vasculitis, Moyamoya syndrome, or arterial dissection; (2) a coexisting cardioembolic source (e.g., atrial fibrillation, mitral stenosis, prosthetic valve, Myocardial infarction within 6 weeks, intra‐cardiac clot, ventricular aneurysm, or bacterial endocarditis); (3) a concomitant intracranial aneurysm or any bleeding disorder; (4) a large infarct core, defined as an ASPECTS of <6 points.

For patients who meet the above conditions, the operator told them of the benefits and risks of EVT. Once the patient agreed to take EVT, he or she entered the EVT group. If the patient refused EVT, he or she was in the control group and received basic medicine treatment.

Age, sex, risk factors such as hypertension, diabetes, dyslipidemia, smoking, previous stroke or TIA, baseline NIHSS, and mRS score were analyzed. Perioperative complications, mainly stent thrombosis and symptomatic intracerebral hemorrhage, were observed. The 1‐year recurrence rate of stroke was taken as a prognostic indicator and was measured by clinical symptoms and diffusion‐weighted imaging (DWI).

### 2.2 Assessment of collateral circulation

DSA was performed before EVT, and MCA occlusion was defined as no forward flow. The ASITN/SIR grade of MCA was taken to assess the collateral circulation; ASITN/SIR ≥ 3 was defined as moderate‐to‐good collateral circulation.

### 2.3 Endovascular therapy

**FIGURE 2:**
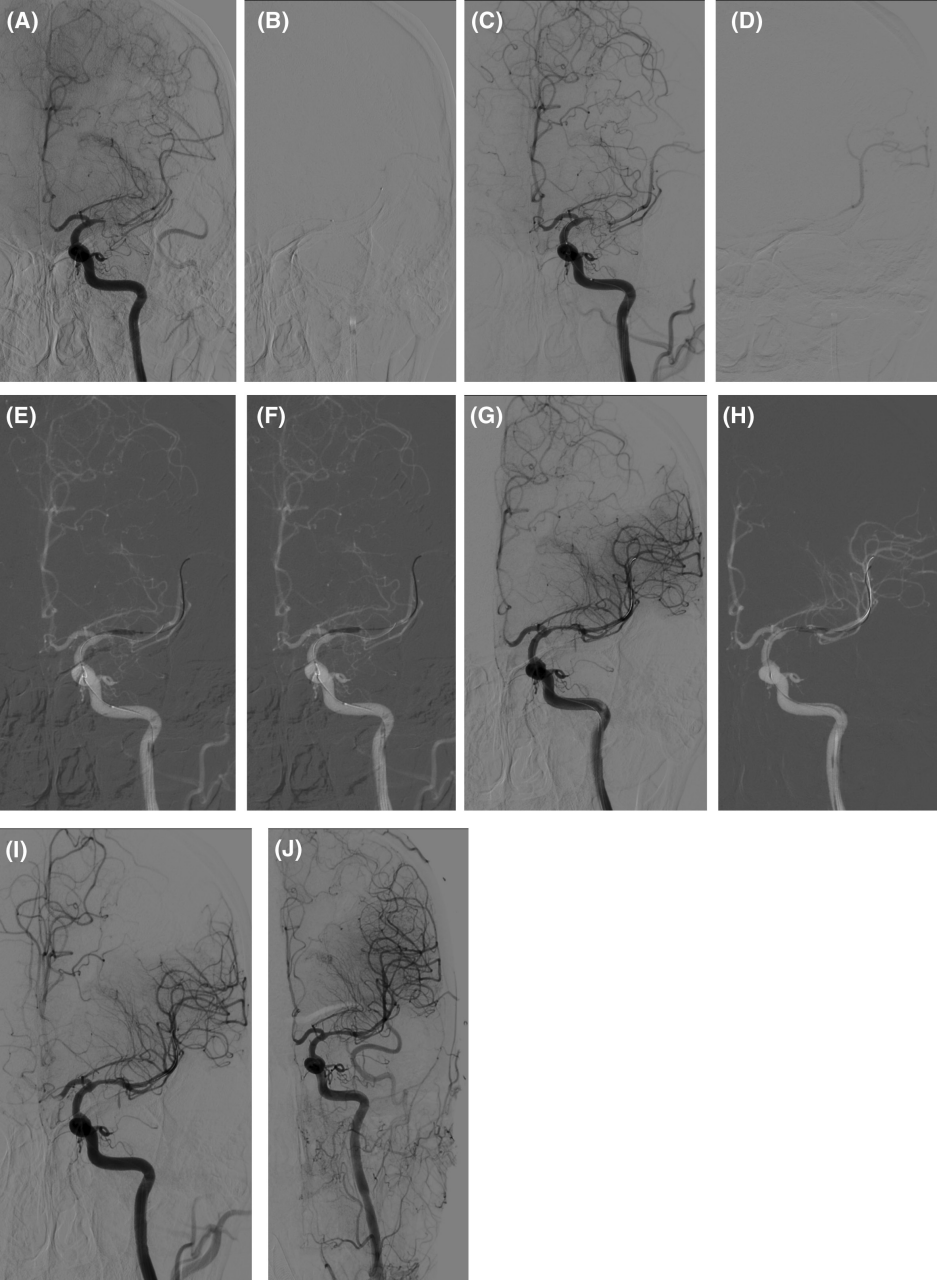
The detailed surgical procedures of endovascular treatment (EVT) for patients with non acute middle cerebral artery occlusion (NAMCAO). (A) The occluded segment was confirmed by digital subtraction angiography (DSA). (B) The microguide wire carries the microcatheter through the occluded segment. (C) The presence of the first pass is confirmed by angiography. (D) The true lumen of the blood vessel by microcathetography, and then change the exchange microguidewire. (E, F) Balloon angioplasty of occluded MCA by 2.0*10 mm sino balloon. (G) Evaluation the effects of balloon angioplasty. (H) Balloon angioplasty of residual stenosis. (I) Angiogram obtained after deployment of a 4.5*22 mm Enterprise stent. (J) Vascular assessment after balloon angioplasty and stenting.

General anesthesia was used for EVT in patients with NAMCAO. The details of the techniques for endovascular recanalization: Sino balloon catheter (Sinomed, China) was taken for balloon angioplasty, and Enterprise stent system (JNJ, USA) was used for stenting. Please refer to Figure 2 for detailed surgical procedures.

### 2.4 Basic medicine therapy

All NAMCAO patients received standard medicine treatment of aspirin (100 mg/day), clopidogrel (75 mg/day), atorvastatin (20 mg/day), or rosuvastatin (10 mg/day).

### 2.5 Outcome measures

The Modified Thrombolysis in Cerebral Ischemia Scale (mTICI) was taken to assess reperfusion status, and an mTICI score of 2b or 3 was considered successful recanalization. Patients who underwent EVT were taken for follow‐up brain CTA or MRA within 4 h to 7 days after endovascular therapy to evaluate the cerebral hemorrhage and the patency of the target vessel. Early reocclusion was defined as the discrete discontinuation of the arterial contrast column within the treated artery on follow‐up CTA or MRA. Symptomatic hemorrhage was defined as hemorrhage associated with neurological deterioration (>3 points increase in the NIHSS). Clinical outcomes were assessed by a stroke neurologist using the mRS score and the NIHSS score during an outpatient visit 90 days after endovascular recanalization. And the recurrence rate in 90 days of stroke was taken as the main prognostic indicator.

### 2.6 Statistical analysis

Continuous variables were described by median and interquartile range. The count (*n*) and percentage (%) were used to describe categorical variables. Baseline and procedural characteristics and treatment outcomes of the EVT group and the control group were compared. The categorical and binary variables were analyzed by χ^2^ test, and the continuous variables were analyzed by the Mann‐Whitney *U* test. Data that did not exhibit a normal or Gaussian distribution was analyzed via a nonparametric equivalent. All statistical analyses were completed by SPSS software (version 25.0; IBM SPSS, Chicago, IL), and a *p* value <0.05 was considered significant.

## 3 RESULTS

### 3.1 Baseline characteristics

Table 1 showed that our study included 98 patients with symptomatic nonacute middle cerebral artery occlusion (78.6% male; median age 57). Of these, 56 patients received endovascular treatment. The baseline and procedural characteristics of patients are presented in Table 1. Overall, 60.2% (59/98) of patients had hypertension, 35.7% (35/98) had diabetes, 38.8% (38/98) had a smoking history, 10.2% (10/98) had hyperlipidemia, and 33.7% (33/98) had a history of prior ischemic stroke. The baseline mRS and NIHSS scores showed no difference between the two groups. And all patients have received standard antiplatelet medication.

**TABLE 1:** Comparison of baseline and procedural characteristics between patients who received EVT and control.

	All patients (*n* = 98)	No EVT (*n* = 42)	EVT (*n* = 56)	*p* value
Age, years	57 (50.0–67.0)	56.0 (50.5–66)	57 (50.0–68.75)	0.586
Sex, male	77 (78.6)	32 (76.2)	45 (80.4)	0.6189
Risk factors				
Hypertension	59 (60.2)	22 (52.4)	37 (66.7)	0.171
Diabetes	35 (35.7)	15 (35.7)	20 (35.7)	1.0
Smoking	38 (38.8)	15 (35.7)	23 (41.1)	0.590
Dyslipidemia	10 (10.2)	3 (7.1)	7 (12.5)	0.386
Previous stroke or TIA	33 (33.7)	13 (31.0)	20 (35.7)	0.622
Baseline NIHSS score	3 (3–6)	3 (2–5.25)	3 (2–6)	0.34
Baseline mRS score	2 (1–2)	2 (1–2)	2 (1–2)	0.80
Baseline ASITN/SIR	3 (3–4)	3 (3–4)	3 (3–4)	0.28
Postprocedural oral antiplatelet medication	98 (100)	42 (100)	56 (100)	1

*Abbreviations*: EVT, endovascular treatment; mRS, Modified Rankin Scale; NIHSS, National Institutes of Health Stroke Scale; TIA, transient ischemic attack.

### 3.2 Perioperative outcome

The perioperative outcome is shown in Table 2. 94.6% patients (53/56) achieved successful reperfusion after endovascular therapy (TICI = 3). The perioperative complication rate was 7.1% (4/56). Two patients (3.6%) experienced symptomatic hemorrhage. The mortality rate was 1.8% (1/56), and the patient died of symptomatic hemorrhage. All patients received oral antiplatelet medication after endovascular recanalization. Table 2 presents the detailed clinical and angiographic outcomes. One patient experienced vessel perforation with subarachnoid hemorrhage during attempts to traverse the occluded segment with a microwire, and fall to recanalization. Another two patients fell to recanalization, because the microwire could not pass the occlusion site. Two patients showed symptomatic ICH (NIHSS rise ≥4) after successful recanalization, of which one patient showed hyperperfusion. One patient showed branch embolization (ipsilateral anterior cerebral artery). One patient showed stent thrombosis within 24 h after EVT. Three patients (6.3%) showed contrast agent exudation, and six patients (12.5%) showed asymptomatic hemorrhage in dyna‐CT and follow‐up CT, and the hemorrhage was absorbed in 7 days with no symptoms left.

**TABLE 2:** Perioperative outcome of patients received EVT.

	EVT (*n* = 56)
Successful reperfusion	53 (94.6)
Postprocedural perfusion	
TICI = 3	53 (94.6)
TICI = 2b	0 (0)
TICI = 0	3 (5.4)
Complication rate	4 (7.1)
Symptomatic ICH	2 (3.6)
Branch embolization	1 (1.8)
Stent thrombosis	1 (1.8)
Death	1 (1.8)

*Abbreviation*: EVT, endovascular treatment.

### 3.3 Follow‐up outcome

All patients received clinical and imaging follow‐up for more than 3 months. Table 3 showed that stroke occurred at a rate of 12.1% (12/98) during follow‐up, 8 patients (21.4%) in control group, and 4 patients (7.1%) in the EVT group. The 90‐day NIHSS and mRS scores showed no statistical difference between the two groups. In Table 4, we excluded patients who could not recanalize, patients with complications, and statistics again. Stroke occurred at a rate of 6.1% (three patients) in the EVT group. Also, the 90‐day NIHSS and mRS scores showed no statistical difference between the two groups.

**TABLE 3:** Comparison of treatment outcomes between patients who received EVT and control.

	All patients (*n* = 98)	No EVT (*n* = 42)	EVT (*n* = 56)	*p* value
Stroke recurrence	12 (12.1)	8 (21.4)	4 (7.1)	0.03
mRS score at discharge	2 (1–2)	2 (1–2)	2 (1–2)	0.72
NIHSS score at discharge	2.5 (1–4)	3 (2–4)	2 (2–5)	0.73
90‐day mRS score	1 (1–2)	2 (1–2)	1 (1–2)	0.37
90‐day NIHSS score	2 (1–4)	2 (2–3)	2 (1–4)	0.14
90‐day mRS score <3	90 (91.8)	39 (92.9)	51 (91.1)	0.30
Complication rate	4 (4.1)	0 (0)	4 (7.1)	0.13
Death	1 (1.0)	0 (0)	1 (1.8)	0.99

*Abbreviations*: EVT, endovascular treatment; NIHSS, National Institutes of Health Stroke Scale; mRS, Modified Rankin Scale.

**TABLE 4:** Comparison of treatment outcomes between patients who received EVT and control after adjustment.

	All patients (*n* = 91)	No EVT (*n* = 42)	EVT (*n* = 49)	*p* value
Stroke recurrence	11 (12.1)	8 (21.4)	3 (6.1)	0.03
90‐day mRS score	1 (1–2)	1 (1–2)	1 (1–2)	0.79
90‐day NIHSS score	1 (2–3)	2 (2–3)	1 (2–3.5)	0.55
90‐day mRS score <3	90 (91.8)	39 (92.9)	46 (93.9)	0.42

*Abbreviations*: EVT, endovascular treatment; mRS, Modified Rankin Scale; NIHSS, National Institutes of Health Stroke Scale; TIA, transient ischemic attack.

## 4 DISCUSSION

Our study was to analyze the safety and effectiveness of endovascular recanalization for patients with nonacute middle cerebral artery occlusion. We enrolled a total of 56 surgical patients. And we found that EVT seems to reduce the occurrence of stroke in patients with NAMCAO. For safety, the success rate was 94.6%, and the complication rate was 7.1%. Two patients showed symptomatic ICH, during which one patient presented hyperperfusion, and the hemorrhage of another patient might be related to a surgical operation, of which one patient died. In the follow‐up study, we will use TCD to measure the cerebral blood flow velocity and use noninvasive intracranial pressure monitoring to measure the hyperperfusion after EVT.

In our study, we found that EVT seemed to reduce the recurrence rate of stroke in patients with NAMCAO. However, since the baseline mRS of all patients who received EVT was between 0 and 3, the 90‐day mRS and NIHSS score showed no difference between the two groups. Also, when we ruled out patients who could not recanalize and patients with complications, the outcome still showed no statistical difference. And the NIHSS and mRS showed no significant difference before and after successful recanalization. So EVT could only reduce the recurrence rate of stroke in patients with NAMCAO?

**FIGURE 3:**
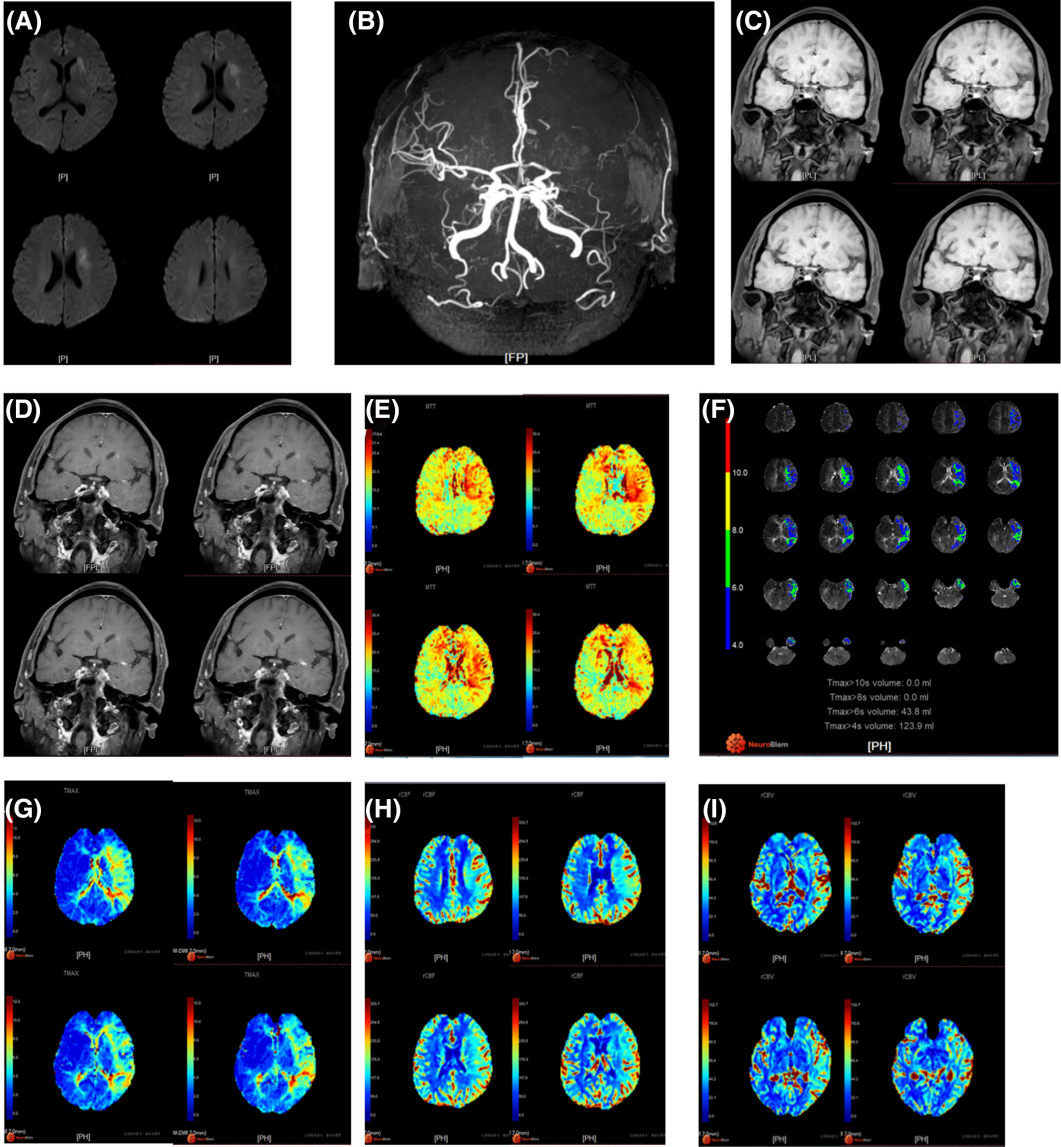
MRA, High‐resolution Vessel Wall and Magnetic Resonance perfusion measurement of non acute middle cerebral artery occlusion (NAMCAO) patients. (A) DWI showed the cerebral infarction of the patient. (B) MRA showed the occluded Left‐MCA of the patient. (C, D) High‐resolution vessel wall of the patient showed the occlusion site and length. MTT (E), Tmax (F, G), CBF (H), CBV (I) showed the perfusion condition of the patient.

In this study, we do not aim to recanalize the occluded middle cerebral artery for all patients, but we try to select NAMCAO patients who could benefit from EVT. The main cause of stroke reoccurrence in NAMCAO patients is hypoperfusion; restoring forward blood flow with the help of EVT could theoretically improve perfusion and reduce stroke recurrence. We found that patients with NAMCAO who meet the following conditions were more likely to benefit from EVT: (1) decreased perfusion in the occluded arterial supply area (measured by magnetic resonance perfusion); (2) short occlusion length (less than 2 cm, measured by high‐resolution vessel wall); (3) occlusion site only involves the horizontal segment of the middle cerebral artery. And the measurement before EVT is shown in Figure 3. The intracranial hemorrhage in the perioperative period is usually related to two factors: violent operations and hyperperfusion. So we recommend that this surgery be performed in a senior stroke center by an experienced neurointerventional physician. And blood pressure management and intracranial pressure monitoring after EVT could reduce the hemorrhage caused by hyperperfusion.^21^, ^22^ It has been reported that clinical classification could affect the prognosis. Proffers Gao feng tried to divide NAMCAO into 3 categories based on anatomical structure and found Type I and Type II NAMCAO patients were more likely to benefit from EVT.^23^

Still, there are several limitations to our study. This study is a single‐center retrospective study. The measurement methods for good outcomes are relatively simple; only 90‐day mRS reverse and 90‐day NIHSS reverse were considered. The perfusion of hypoperfused brain tissue after endovascular treatment was not measured. And the cognitive function of patients was lacking. In the future, multiple‐center randomized controlled trials are needed to investigate whether endovascular treatment is effective and safe for NAMCAO patients.

## 5 CONCLUSION

Among patients with symptomatic nonacute middle cerebral artery occlusion with a short length of occlusion and moderate‐to‐good collateral circulation, endovascular treatment seemed to be safe. And endovascular treatment seemed to reduce the recurrence rate of stroke in these patients.

## CONFLICT OF INTEREST STATEMENT

Xu, Yun is an Editorial Board member of CNS Neuroscience and Therapeutics and a co‐author of this article. To minimize bias, they were excluded from all editorial decision‐making related to the acceptance of this article for publication.

## Data Availability

The data that support the findings of this study are available from the corresponding author upon reasonable request.
